# Platelet-derived growth factor signaling in pericytes promotes hypothalamic inflammation and obesity

**DOI:** 10.1186/s10020-024-00793-z

**Published:** 2024-02-05

**Authors:** Akira Okekawa, Tsutomu Wada, Yasuhiro Onogi, Yuki Takeda, Yuichiro Miyazawa, Masakiyo Sasahara, Hiroshi Tsuneki, Toshiyasu Sasaoka

**Affiliations:** 1 Department of Clinical Pharmacology, University of Toyama, 2630 Sugitani, Toyama, 930-0194 Japan; 2 Research Center for Pre-Disease Science, University of Toyama, 2630 Sugitani, Toyama, Japan; 3 Department of Pathology, University of Toyama, 2630 Sugitani, Toyama, Japan; 4 Department of Integrative Pharmacology, University of Toyama, 2630 Sugitani, Toyama, Japan

**Keywords:** Inflammation, Microglia, Obesity, Pericytes, Platelet-derived growth factor

## Abstract

**Background:**

Pericytes are a vital component of the blood–brain barrier, and their involvement in acute inflammation was recently suggested. However, it remains unclear whether pericytes contribute to hypothalamic chronic inflammation and energy metabolism in obesity. The present study investigated the impact of pericytes on the pathophysiology of obesity by focusing on platelet-derived growth factor (PDGF) signaling, which regulates pericyte functions.

**Methods:**

Tamoxifen-inducible systemic conditional PDGF receptor β knockout mice (*Pdgfrb*^∆SYS^-KO) and Calcium/calmodulin-dependent protein kinase type IIa (CaMKIIa)-positive neuron-specific PDGF receptor β knockout mice (*Pdgfrb*^∆CaMKII^-KO) were fed a high-fat diet, and metabolic phenotypes before and 3 to 4 weeks after dietary loading were examined. Intracellular energy metabolism and relevant signal transduction in lipopolysaccharide- and/or platelet-derived growth factor-BB (PDGF-BB)-stimulated human brain pericytes (HBPCs) were assessed by the Seahorse XFe24 Analyzer and Western blotting. The pericyte secretome in conditioned medium from HBPCs was studied using cytokine array kit, and its impact on polarization was examined in bone marrow-derived macrophages (BMDMs), which are microglia-like cells.

**Results:**

Energy consumption increased and body weight gain decreased after high-fat diet loading in *Pdgfrb*^∆SYS^-KO mice. Cellular oncogene fos (cFos) expression increased in proopiomelanocortin (POMC) neurons, whereas microglial numbers and inflammatory gene expression decreased in the hypothalamus of *Pdgfrb*^∆SYS^-KO mice. No significant changes were observed in *Pdgfrb*^∆CaMKII^-KO mice. In HBPCs, a co-stimulation with lipopolysaccharide and PDGF-BB shifted intracellular metabolism towards glycolysis, activated mitogen-activated protein kinase (MAPK), and modulated the secretome to the inflammatory phenotype. Consequently, the secretome showed an increase in various proinflammatory chemokines and growth factors including Epithelial-derived neutrophil-activating peptide 78 (C-X-C motif chemokine ligand (CXCL)5), Thymus and activation-regulated chemokine (C–C motif chemokine (CCL)17), Monocyte chemoattractant protein 1 (CCL2), and Growth-regulated oncogene α (CXCL1). Furthermore, conditioned medium from HBPCs stimulated the inflammatory priming of BMDMs, and this change was abolished by the C-X-C motif chemokine receptor (CXCR) inhibitor. Consistently, mRNA expression of CXCL5 was elevated by lipopolysaccharide and PDGF-BB treatment in HBPCs, and the expression was significantly lower in the hypothalamus of *Pdgfrb*^∆SYS^-KO mice than in control *Pdgfrb*^flox/flox^ mice (FL) following 4 weeks of HFD feeding.

**Conclusions:**

PDGF receptor β signaling in hypothalamic pericytes promotes polarization of macrophages by changing their secretome and contributes to the progression of obesity.

**Supplementary Information:**

The online version contains supplementary material available at 10.1186/s10020-024-00793-z.

## Introduction

The central nervous system (CNS) integrates various peripheral signals from each organ to regulate systemic glucose and energy metabolism. Agouti-related protein (AgRP) and proopiomelanocortin (POMC) neurons in the arcuate nucleus (ARC) of the hypothalamus respond to these signals by changing neuronal activity to maintain energy homeostasis (Manceau et al. 2020). The disruption of the regulatory mechanism increases food consumption and decreases energy expenditure in obesity (Waterson and Horvath 2015). Chronic inflammation in the hypothalamus has been suggested as the underlying mechanism in both rodents and humans (Jais et al. 2017). Therefore, the prolonged feeding of a high-fat diet (HFD) activated resident microglia and the infiltration of macrophages in the hypothalamus of mice (Lee et al 2018), whereas the inhibition of microglial nuclear factor-κB (NF-κB) signaling markedly suppressed diet-induced hyperphagia and weight gain (Valdearcos et al. 2017). Moreover, microglial activation occurred even with short-term HFD feeding in the hypothalamus of mice (Folick et al. 2022); however, the mechanisms that trigger microglial activation in the early stage of obesity remain unclear.

The blood–brain barrier (BBB) is crucial for protecting parenchymal neurons by limiting neurotoxic factors in the CNS (Segarra et al. 2021). Pericytes are a vital component of vascular cells in the BBB, they cover endothelial cells to stabilize blood vessels, and they contribute to the maintenance of BBB functions and microvessel maturation under physiological states (Sweeney et al. 2016). In addition, pericytes play a crucial role in regulating cerebral blood flow (Brown et al. 2019). In adults, platelet-derived growth factors (PDGF)-BB secreted from endothelial cells acts on PDGF receptor β (PDGFRβ) that is almost restrictively expressed in pericytes and vascular smooth muscle cells (VSMCs) (Sil et al 2018; Vanlandewijck et al. 2018). Signaling between endothelial PDGF-BB and pericyte PDGFRβ is known to play an essential role in maintaining vascular function. Thus, congenital impairment of PDGFRβ signaling in *Pdgfrb*^±^ mice or *Pdgfb*^ret/ret^ mice lacking the retention motif of PDGF-BB have been reported to exhibit pericyte loss and BBB dysfunction (Crouch et al. 2023).

Emerging evidence suggests that pericytes possess functional heterogeneity and regulate several aspects of immune responses, including the extravasation of leukocytes and polarization of inflammatory cells in the CNS (Sweeney et al. 2018; Rustenhoven et al. 2017). Pericytes can phagocytose other cells and present antigens to immune cells (Brown et al. 2019). Notably, they function as initial sensors of systemic inflammation in the brain and maintain vascular stability to prevent neurodegenerative diseases, such as Alzheimer’s disease (Duan et al. 2018; Smyth et al. 2022). Moreover, PDGF-BB has been shown to regulate pericyte responses under pathological conditions in vitro (Gaceb et al. 2018). Based on these findings, we hypothesized that PDGF signaling may be involved in the development of chronic inflammation in the hypothalamus.

We herein report a novel pathological role for pericytes in obesity. Hypothalamic inflammation after feeding with a high-fat diet (HFD) was attenuated in systemic *Pdgfrb*-deficient mice, which increased energy expenditure and decreased body weight gain. The secretome profile of cultured human brain pericytes (HBPCs) indicated the importance of the chemokine communications as the pathological link between pericytes and microglia/macrophage in the hypothalamus in the early stage of diet-induced obesity, which was driven by PDGF signaling in pericytes.

## Materials and methods

### Animals

*Pdgfrb*^flox/flox^ mice (FL) (Gao et al. 2005) on the C57BL/6 J genetic background were crossed with Cre-estrogen receptor transgenic mice (The Jackson Laboratory, JAX stock #004682). Their offspring, Cre-ER; *Pdgfrb*^flox/flox^, and littermate controls were orally administered 225 mg/kg tamoxifen (T006000, Toronto Research Chemicals, Canada) for 5 consecutive days at 8–9 weeks old to produce conditional systemic *Pdgfrb* knockout mice (*Pdgfrb*^∆SYS^-KO) (Onogi et al. 2017). FL were crossed with Calcium/calmodulin-dependent protein kinase type II (CaMKIIa)-Cre transgenic mice (kindly gifted from the RIKEN BioResource Research Center) to generate CaMKIIa-positive neuron-specific *Pdgfrb* knockout mice (*Pdgfrb*^∆CaMKII^-KO) (Shioda et al. 2012). Mice were housed under a controlled temperature (20–26℃) and 12-h light–dark cycle with free access to water and a normal chow diet (PicoLab Rodent Diet, LabDiet, USA). Male mice were used for each experiment. Mice were fed a 60 kcal% HFD (D12492; Research Diets, USA) from 10–11 weeks old for the indicated period. Energy expenditure and the locomotor activity in mice were assessed using metabolic chambers (MK-5000RQ; Muromachi Kikai, Japan), and energy expenditure (Kcal/min/Kg^0.75^) was calculated using the following formula: (1.07 × VO_2_/VCO_2_ + 3.98) × VO_2_ / (body weight)^0.75^, according to the manufacture’s instrument. Food consumption by each mouse for 24 h was measured in isolated cages before and 3 weeks after HFD feeding. Mice were euthanized after 4 weeks of HFD feeding for further biological analyses.

### Cell cultures

HBPCs purchased from Applied Cell Biology Research Institute (Kirkland, USA) were maintained on collagen I-coated dishes (50 μg/mL) with CS-C Complete Medium Kit R (Cell Systems, USA) or Dulbecco’s Modified Eagle Medium/Ham’s F-12 (DMEM/F-12: Thermo Fisher Scientific, USA) containing 10% fetal bovine serum (FBS) supplemented with 2 mM L-alanyl-L-glutamine. An antibiotic–antimycotic mixed stock solution was added to both media. Medium was changed every 2–3 days and cells were passaged every week (Watanabe et al. 2020). Bone marrow-derived macrophages (BMDMs) were differentiated from the bone marrow cells of C57BL/6J mice in RPMI 1640 media, as previously described (Wada et al. 2017). Cells were treated with 10 or 100 ng/mL lipopolysaccharide (LPS: L6529, Sigma, USA) and/or 100 ng/mL recombinant human PDGF-BB (PDGF-BB: 577302, BioLegend, USA) in serum-free medium. Bovine serum albumin (BSA)-conjugated palmitate was prepared by mixing fatty acid free, low endotoxin-BSA (A8806, Sigma) with sodium palmitate (P9767, Sigma) at a ratio of 6:1 (Ono-Moore et al.^2018^). HBPCs were treated with 100 μM BSA-conjugated palmitate and/or 100 ng/mL PDGF-BB in serum-free medium for 6 h. Rat primary microglia were prepared from E17-18 embryos from pregnant Sprague–Dawley rats. The cortical tissues from 5–7 embryos were dispersed in DMEM containing 10% FBS and the cortical cells in 5 μg/mL poly-D-lysine-coated 24-well plates (5 × 10^5^ cells per well) were cultured for 14 days to obtain microglia. Purity of microglia was approximately 50% by immunostaining with Iba1.

### Preparation of conditioned medium (CM) and its inhibition assay

HBPCs were seeded at 4 × 10^5^ cells per well in collagen I-coated 12-well plates and grown in DMEM/F-12 medium containing 10% FBS. After a 6-h incubation with serum-free low-glucose DMEM, cells were treated with 100 ng/mL LPS and/or 100 ng/mL PDGF-BB for 1 h. Cells were washed with phosphate-buffered saline (PBS) to remove these stimulants, cultured in fresh low-glucose DMEM for 24 h, and CM was collected and stored at − 80℃ until used. Cytokine release from pericytes in CM was analyzed with the Proteome Profiler Human XL cytokine array kit (ARY022B, R&D Systems, USA). In the inhibition assay of pericyte-derived factors, BMDMs were pretreated with 1 μM SB225002, a C-X-C motif chemokine receptor (CXCR)1/2 antagonist (13336, Cayman), 5 μM RS102895, a C–C motif chemokine (CCR)2 antagonist (ab120812, Abcam), or 100 nM C-021, a CCR4 antagonist (21885, Cayman) for 1 h, followed by a treatment with each CM for 12 h. Cells were then stimulated with 10 ng/mL LPS for 24 h.

### Quantitative real-time PCR

Hypothalami isolated from mice were placed in RNA Later (QIAGEN, USA). Total RNA from the tissues of mice and cultured cells was extracted with TRIsure (NIPPON Genetics, Japan) and subjected to reverse transcription using the ReverTra Ace qPCR RT kit with gDNA remover (TOYOBO, Japan). Relative gene expression was measured by Mx3000/Mx3005P (Agilent Technologies, USA) or CronoSTAR96 (Takara Bio, USA) with Brilliant III Ultra-Fast SYBR Green QPCR Master Mixes (Agilent Technologies) or THUNDERBIRD Next SYBR qPCR Mix (TOYOBO) and 0.2 μM of primers. The sequences of each primer are listed in Additional file 1: Table S1. Gene expression was normalized with 18S ribosomal RNA (Tanaka et al. 2021).

### Western blotting

HBPCs were serum starved and stimulated with 100 ng/mL LPS and/or 100 ng/mL PDGF-BB for 3 h. Harvested cell lysates were subjected to a Western blot analysis, as previously described (Onogi et al. 2020). Antibodies utilized in the present study are listed in Additional file 1: Table S2.

### Immunostaining

Anesthetized mice were perfused with saline and 4% paraformaldehyde (PFA), and tissues were post-fixed with the same fixative overnight at 4 °C and placed in 30% sucrose. The brain and brown adipose tissue (BAT) were sliced at a thickness of 30 μm using a cryostat (CM 3050SIV, Leica, Germany). Brain slices were incubated with 0.2% polyoxyethylene(10) octylphenyl ether (Triton X-100, Wako Pure Chemical, Japan) and 5% BSA (A9647, Sigma) in PBS at 21–24℃ for 1 h. Primary microglia were fixed and dehydrated with a mixture of 4% PFA and 4% sucrose at 21–24℃ for 20 min. BAT slices and primary microglia were permeabilized by 0.3% Triton X-100 in PBS for 30 min and then blocked with protein block (Agilent Technologies) at 21–24℃ for 1 h. Samples were incubated with the primary antibodies at 4℃ overnight. The next day, samples were treated with the secondary antibodies at 21–24℃ for 1 h. Fluorescent images were captured using the confocal microscope system LSM700/900 (Zeiss, Germany) and analyzed by ImageJ/Fiji software (NIH, USA). The number of microglia in the hypothalamic ARC and ventromedial hypothalamic nucleus (VMH) was determined manually by counting the number of Iba1-positive cells in the corresponding bilateral areas of the section.

### Vascular leakage assay using Evans blue

The vascular permeability of the mouse brain was analyzed, as previously reported (Alluri et al. 2016). In brief, Evans blue dye (056-04061, Wako Pure Chemical) was administered intravenously to *Pdgfrb*^∆SYS^-KO and FL mice fed HFD for 4 weeks (2% Evans blue in saline, 4 mL/kg). After 2 h, mice were perfused with saline followed by 4% PFA under anesthesia. Mouse brains were post-fixed overnight in the same fixative and placed in 30% sucrose. Thirty-micrometer-thick brain slices were prepared using a cryostat and incubated with Hoechst 33342. Fluorescent signals were captured using LSM900.

### Measurement of cellular metabolism using Seahorse XFe

HBPCs were seeded on collagen I-coated cell culture plates at 2 × 10^4^ cells per well and cultured overnight in DMEM/F-12 medium containing 10% FBS. Medium was replaced to 500 μL XF DMEM medium containing 10 mM glucose, 1 mM pyruvate, and 2 mM glutamine. Cells were stimulated with 100 ng/mL LPS and/or 100 ng/mL PDGF-BB at 37℃ for 1 h with atmospheric CO_2_. The oxygen consumption rate (OCR) and extracellular acidification rate (ECAR) were measured by the Seahorse XFe24 Analyzer using the Mito Stress Test Kit (Agilent Technologies). During assays, the following compounds were added to cells: 1.5 μM oligomycin, 2 μM carbonyl cyanide-4 (trifluoromethoxy) phenylhydrazone, and a mixture of 0.5 μM rotenone/antimycin A. The assay was performed and data were analyzed by Wave software (ver. 2.6.1).

### Re-analysis of RNA-seq data

Fastq files were downloaded from the SRA database in NCBI (SRP348249). Reads with phred scores < 33 and < 50 bp were removed using fastp (ver. 0.20.1). Quality-checked reads were aligned to Homo_sapiens.GRCh38.dna.primary_assembly using HISAT2 (ver. 2.2.0). Aligned reads were counted using ‘featurecounts’ (ver. 2.0.1). Differential gene expression was analyzed using DEseq2 from iDEP (ver. 96). Cut-off values of fold changes > 1.5 and FDR < 0.05 were used to select differentially expressed genes between two groups (Tsuneki et al. 2022).

### Statistical analysis

Data are presented as the mean ± standard error of the mean (SEM). Statistical analyses were performed with the Student’s *t*-test between two groups. Differences between multiple conditions were evaluated using a one- or two-way ANOVA with Tukey’s test for multiple comparisons by GraphPad Prism 9 software (GraphPad Software Inc., USA). *p < 0.05 and **p < 0.01 indicated significant differences.

## Results

### HFD-induced body weight gain was attenuated by the deletion of PDGF signaling

**Fig. 1:**
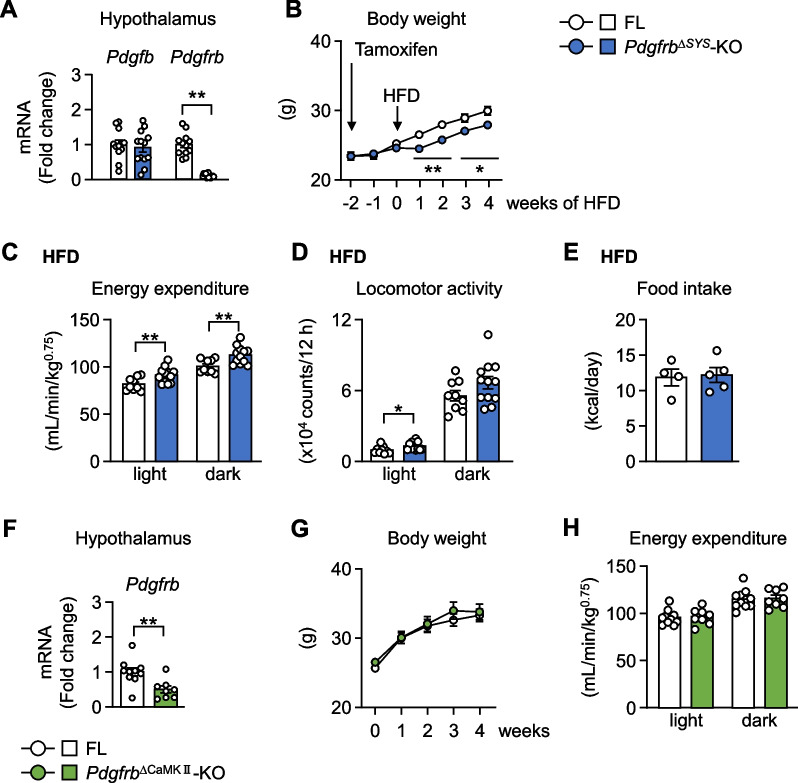
PDGF signaling in pericytes, but not in neurons, mediates the dysfunction of energy metabolism. **A** *Pdgfb* and *Pdgfrb* mRNA levels in the hypothalamus of *Pdgfrb*^∆SYS^-KO fed HFD for 4 weeks (n = 12–13). **B** Body weights of *Pdgfrb*^∆SYS^-KO (n = 12–13). **C–E** Metabolic parameters of *Pdgfrb*^∆SYS^-KO fed HFD for 3–4 weeks. Energy expenditure (**C**), locomotor activity (**D**) (n = 9–12), and food intake (**E**) (n = 4–5). **F** *Pdgfrb* mRNA levels in the hypothalamus of *Pdgfrb*^∆CaMKII^-KO fed HFD for 4 weeks (n = 8–10). **G** Body weights of *Pdgfrb*^∆CaMKII^-KO (n = 8–10). **H** Energy expenditure in *Pdgfrb*^∆CaMKII^-KO fed HFD for 3 weeks (n = 8–10). Data are presented as the mean ± SEM. *p < 0.05 and **p < 0.01

The hypothalamic vasculature is well developed for sensing systemic information via blood flow, and PDGF signaling in pericytes contributes to the regulation of pericyte functions and stabilization of the vasculature (Sweeney et al. 2016). Since metabolic regulation in the hypothalamus is impaired in obesity (Jais and Brüning 2017), we examined the metabolic phenotypes of *Pdgfrb*^∆SYS^-KO before and after 3 weeks of HFD feeding. A marked decrease in *Pdgfrb* expression and no change in *Pdgfb* expression were observed in the hypothalamus of *Pdgfrb*^∆SYS^-KO, indicating efficient Cre-mediated recombination in *Pdgfrb*^∆SYS^-KO (Fig. 1A). We administered tamoxifen to FL and *Pdgfrb*^∆SYS^-KO, and fed them the normal chow diet for 2 weeks, followed by HFD. The body weights of FL and *Pdgfrb*^∆SYS^-KO were similar when mice were maintained on the normal chow diet. However, the body weights of *Pdgfrb*^∆SYS^-KO were significantly lower after HFD feeding for only one week and thereafter (Fig. 1B). After HFD feeding for 4 weeks, the weights of epididymal white adipose tissue (eWAT), inguinal white adipose tissue (iWAT), and BAT were significantly lower in *Pdgfrb*^∆SYS^-KO than in FL (Additional file 1: Fig. S1A). Consistent with body and tissue weight changes, energy expenditure and locomotor activity remained unchanged before HFD feeding, but were significantly higher in *Pdgfrb*^∆SYS^-KO after 3 weeks of HFD feeding (Fig. 1C, D, Additional file 1: Fig. S1B, C). In contrast, food intake did not change between genotypes on both diets (Fig. 1E, Additional file 1: Fig. S1D). These results suggest that PDGF signaling plays a crucial role in the regulation of energy metabolism from the early stage of HFD feeding.

PDGFRβ is almost exclusively expressed in pericytes/VSMCs, but is also expressed in some neurons (Sil et al. 2018; Crouch et al. 2023). Therefore, we generated *Pdgfrb*^∆CaMKII^-KO mice to clarify the impact of PDGFRβ signaling in CaMKIIa-positive neurons on energy metabolism during HFD feeding. *Pdgfrb* expression significantly decreased in the hypothalamus of *Pdgfrb*^∆CaMKII^-KO (Fig. 1F), suggesting the deletion of CaMKIIa-positive neuron-selective expression. However, neither body weight nor energy expenditure changed in *Pdgfrb*^∆CaMKII^-KO upon HFD feeding (Fig. 1G, H). These results indicate that PDGF signaling in pericytes, but not CaMKIIa-positive neurons, is responsive to decreased energy expenditure in HFD-induced obesity.

### PDGF signaling mediates microglial activity in obese mice

**Fig. 2:**
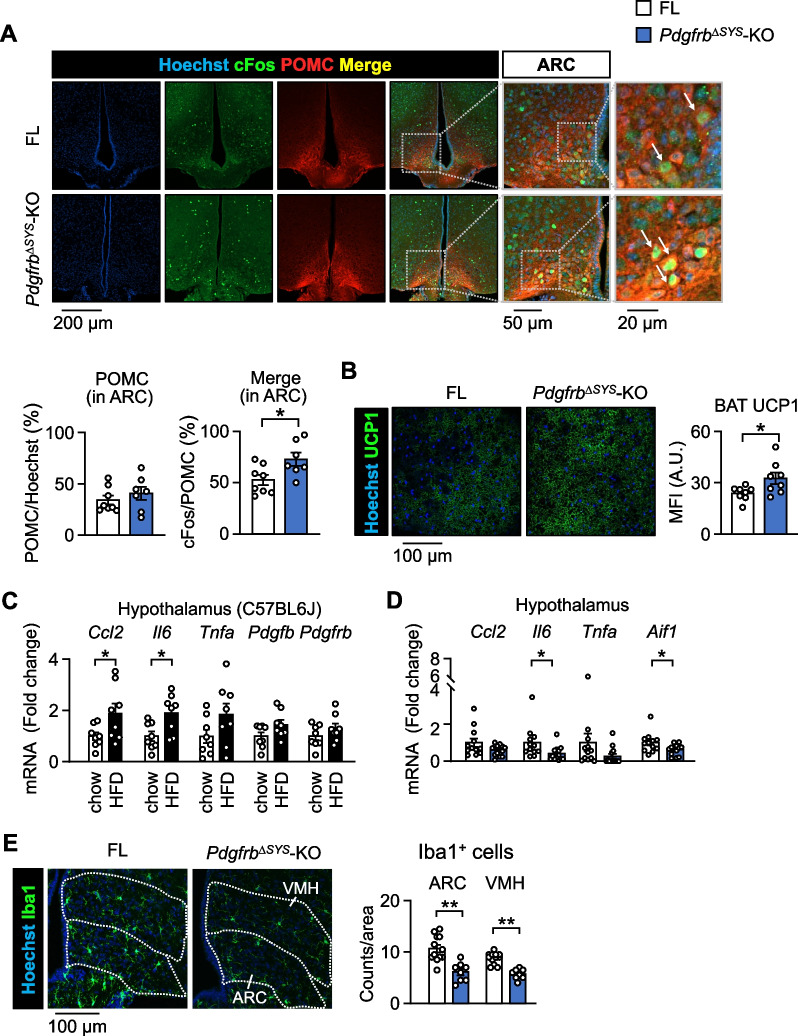
PDGF signaling mediates chronic inflammation in the hypothalamus. **A** Representative confocal images of cFos (green) and POMC (red) immunoreactivities and counterstaining with Hoechst 33342 (blue) in the ARC of FL and *Pdgfrb*^∆SYS^-KO fed HFD for 4 weeks (n = 7–8). Scale bar = 200 μm. The right two panels are high magnification of the gray line square area. White arrows indicate Hoechst^+^cFos^+^POMC^+^ cells. Scale bar = 50 or 20 μm. The ratio of POMC-positive neurons in Hoechst-positive cells and cFos-POMC double-positive neurons in POMC-positive neurons are quantified. **B** Representative confocal images of UCP1 immunoreactivity (green) and Hoechst 33,342 (blue) staining in the BAT of FL and *Pdgfrb*^∆SYS^-KO fed HFD for 4 weeks. Scale bar = 100 μm. Mean fluorescent intensity (MFI) of UCP1 in the BAT (n = 8). **C** mRNA levels in the hypothalamus of C57BL6J mice fed a normal chow diet or HFD for 4 weeks (n = 8). **D** mRNA levels in the hypothalamus of FL and *Pdgfrb*^∆SYS^-KO fed HFD for 4 weeks (n = 12–13). **E** Representative confocal images of Iba1 immunoreactivity (green) in the ARC and VMH of FL and *Pdgfrb*^∆SYS^-KO fed HFD for 4 weeks (n = 8–12). Scale bar = 100 μm. Each region was indicated by a dotted line. The numbers of Iba1-positive microglia in the ARC and VMH were quantified. Data are presented as the mean ± SEM. *p < 0.05 and **p < 0.01

Hypothalamic POMC neurons play a significant role in the regulation of energy metabolism (Tran et al. 2022). Therefore, we examined POMC neuronal activity by immunostaining with cellular oncogene fos (cFos) in the ARC of *Pdgfrb*^∆SYS^-KO after 4 weeks of HFD feeding (Fig. 2A). Although the total number of POMC neurons did not change, the percentage of cFos-positive POMC neurons was significantly higher in *Pdgfrb*^∆SYS^-KO than in FL. The protein intensity of uncoupling protein 1 (UCP1) in BAT, a downstream thermogenic effector of POMC neurons via sympathetic activation (Tran et al. 2022), was consistently higher in *Pdgfrb*^∆SYS^-KO (Fig. 2B). In contrast, neither the expression of *Ucp1* in iWAT nor *UCP3* in the soleus muscles changed in FL and *Pdgfrb*^∆SYS^-KO on HFD (Additional file 1: Fig. S1E, F).

We then investigated chronic inflammation in the hypothalamus of HFD-fed mice. *Ccl2* and *Il6* expression significantly increased, whereas *Pdgfb* and *Pdgfrb* expression remained unchanged in the hypothalamus of C57BL/6J mice fed HFD for 4 weeks (Fig. 2C). Importantly, after 4 weeks of HFD feeding, the expression of the inflammatory cytokines *Il6* and *Aif1* encoding the microglial marker Iba1 in the hypothalamus were significantly lower in *Pdgfrb*^∆SYS^-KO than in FL (Fig. 2D). Under this condition, the number of Iba1-positive microglia was significantly lower in both the ARC and VMH of *Pdgfrb*^∆SYS^-KO (Fig. 2E). In contrast, the expression of proinflammatory genes in eWAT did not change between *Pdgfrb*^∆SYS^-KO and FL fed HFD for 4 weeks (Additional file 1: Fig. S1G). Therefore, enhanced POMC neuronal activity and decreased inflammation in the hypothalamus of *Pdgfrb*^∆SYS^-KO were observed independently of obesity-associated chronic inflammation in adipose tissue.

The congenital impairment of PDGFRβ signaling is known to cause vascular dysfunction, such as extravasation, which may affect the development of chronic inflammation (Crouch et al. 2023). Therefore, we injected Evans blue dye into the tail vein and examined vascular leakage in the brain of tamoxifen-inducible *Pdgfrb*^∆SYS^-KO. No obvious extravasation of the dye was noted in macroscopic brain or microscopic hypothalamic sections from *Pdgfrb*^∆SYS^-KO or FL fed HFD for 4 weeks (Additional file 1: Fig. S2). These results suggest that PDGFRβ signaling in pericytes regulates hypothalamic inflammation, resulting in the suppression of POMC neuronal activity and decreased BAT thermogenesis in adult mice during 4 weeks of HFD feeding without affecting vascular barrier function.

### Pericytes mediate microglial activation

**Fig. 3:**
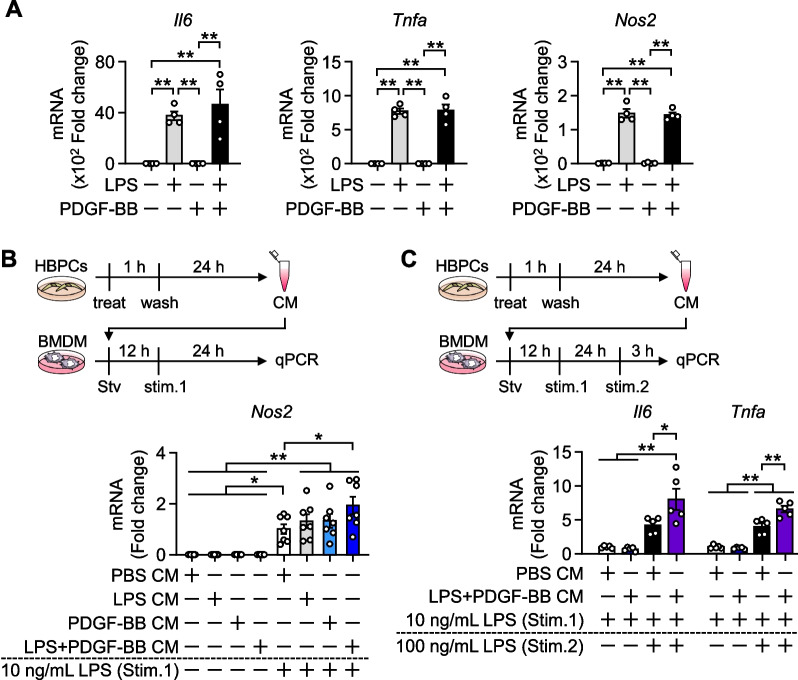
HBPC-derived conditioned medium induces the inflammatory polarization of BMDMs. **A** *Il6*, *Tnfa*, and *Nos2* mRNA levels in BMDMs treated with 100 ng/mL LPS and/or 100 ng/mL recombinant human PDGF-BB for 3 h (n = 4). **B** Upper; timeline of the experiment. Lower; *Nos2* mRNA level in BMDMs (n = 7). HBPCs were treated with LPS and/or PDGF-BB for 1 h, and cells were washed and replaced with fresh medium. The conditioned medium (CM) of HBPCs was harvested after 24 h. BMDMs were then treated with each CM in serum-starved medium for 12 h and primed with 10 ng/mL LPS (stim. 1) for 24 h. **C** Upper; timeline of the experiment. Lower; *Il6* and *Tnfa* mRNA levels in BMDMs (n = 5). BMDMs were treated with each CM in serum-starved medium for 12 h, primed with 10 ng/mL LPS (stim. 1) for 24 h, and stimulated with 100 ng/mL LPS (stim. 2). Data are presented as the mean ± SEM. *p < 0.05 and **p < 0.01, significantly different between each group. *Stv* starvation, *stim.* stimulation

We examined the impact of PDGF-BB on the activation of microglia-like cells. The LPS stimulation markedly increased the expression of *Il6*, *Tnfa*, and *Nos2* in BMDMs. In contrast, PDGF-BB alone did not affect their expression, even when co-stimulated with LPS (Fig. 3A), suggesting that PDGFRβ signaling did not directly regulate activation of BMDMs. Microglial activation was weaker in *Pdgfrb*^∆SYS^-KO on HFD (Fig. 2D, E), indicating the presence of inflammation-mediating factors, which were secreted from other cells in response to PDGFRβ signaling. We speculated that pericytes were the mediator cells responsible, presumably via secretory factors, for the link between PDGFRβ signaling in pericytes and microglial activation. To demonstrate this, we collected pericyte CM from HBPCs and examined its impact on inflammatory responses in BMDMs. We treated HBPCs with LPS, PDGF-BB, or LPS and PDGF-BB for 1 h. After washing cells with PBS to remove these factors, we cultured cells in fresh medium for 24 h and harvested media as PBS CM, LPS CM, PDGF-BB CM, and LPS + PDGF-BB CM, respectively. BMDMs were cultured with various CM in the absence or presence of 10 ng/mL LPS to induce their priming. The expression of *Nos2*, an indicator of inflammatory activation in macrophage and microglia, significantly increased in LPS-primed BMDMs cultured with any CM (Fig. 3B, lanes 1–4 vs lanes 5–8). Importantly, the expression was significantly higher in LPS-primed BMDMs cultured with LPS + PDGF-BB CM (lane 8) than with PBS CM (lane 5). We then investigated the impact of pericyte secretory factors on cytokine expression in BMDMs. Naïve BMDMs were primed with 10 ng/mL LPS and pericyte CMs for 24 h (stim. 1) and then stimulated with 100 ng/mL LPS for 3 h (stim. 2). The expression of *Il6* and *Tnfa* was significantly higher in BMDMs primed with LPS + PDGF-BB CM than with PBS CM (Fig. 3C).

Microglia change their morphology depending on their activation and polarization. Therefore, we further investigated the influence of pericytes on the morphology of microglia using Iba1^+^ rat primary microglia. When cells were incubated with LPS + PDGFB CM, the size of cell body significantly increased and the morphology changed from ramified to amoeboid shape (Additional file 1: Fig. S3). These results strongly indicate that pericytes enhance the inflammatory polarization of microglia by producing secretory factors in response to PDGF-BB and inflammatory stimuli.

### PDGF-BB stimulates glycolytic metabolism in HBPCs

**Fig. 4:**
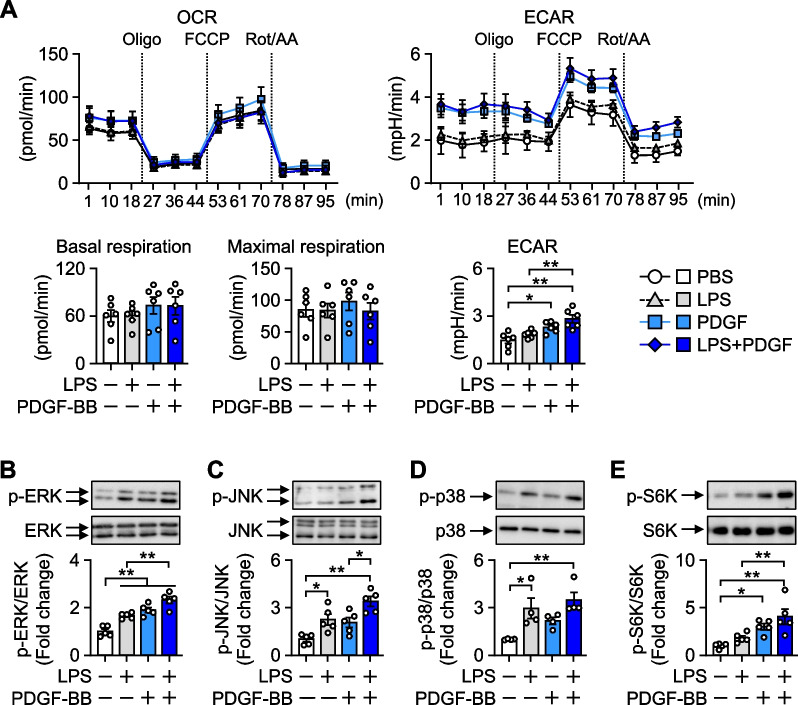
PDGF signaling shifts cellular metabolism towards glycolysis. **A** HBPCs were stimulated with LPS and/or PDGF-BB for 1 h and the oxygen consumption rate (OCR) and extracellular acidification rate (ECAR) were analyzed by a flux analyzer (n = 6). Oligo; oligomycin, FCCP; carbonyl cyanide-4 (trifluoromethoxy) phenylhydrazone, Rot/AA; a mix of rotenone/antimycin A. **B–E** HBPCs were treated with 100 ng/mL LPS and/or 100 ng/mL recombinant human PDGF-BB for 3 h. Representative Western blotting and quantified results are shown (n = 4–5). Data are presented as the mean ± SEM. *p < 0.05 and **p < 0.01, significantly different between each group

We investigated the effects of the PDGF-BB stimulation on the transcriptome of primary HBPCs using public RNA-Seq data (SRP348249). Most of the highly enriched pathways in PDGF-BB stimulated pericytes were associated with inflammation (Additional file 1: Fig. S4). Since changes in intracellular metabolism are closely related to cell functions and cytokine production (Chou et al. 2022), we examined the impact of an LPS and PDGF-BB stimulation on the cellular metabolic pathways involved in energy production in HBPCs using a flux analyzer. Neither LPS nor PDGF-BB affected the OCR, an indicator of mitochondrial respiration. In contrast, PDGF-BB significantly increased the ECAR, indicating enhanced basal glycolysis. The increased ECAR in PDGF-BB-treated pericytes was sustained following a treatment with oligomycin, an ATP synthase inhibitor, and a mixture of rotenone and antimycin A, inhibitors of mitochondrial respiratory chain complexes I and III, respectively. Therefore, PDGF-BB also enhanced mitochondria-uncoupled glycolysis. On the other hand, LPS did not affect the ECAR, at least under our experimental conditions in pericytes (Fig. 4A).

We also investigated the intracellular signaling properties of pericytes following an LPS and PDGF-BB stimulation because they are closely associated with the induction of metabolic reprogramming (Chou et al. 2022; Meng et al 2021). LPS significantly induced the phosphorylation of ERK, JNK, and p38-mitogen-activated protein kinase (MAPK). On the other hand, PDGF-BB stimulated the phosphorylation of ERK, JNK, and S6 kinase, which was consistent with the results of RNA-Seq showing that the MAPK signaling pathway was enriched in PDGF-BB-stimulated pericytes (Fig. 4B–E, Additional file 1: Fig. S4). Therefore, PDGF signaling stimulated MAPK and mammalian target of rapamycin (mTOR) signals, which coupled with the activation of glycolytic metabolism in pericytes.

### Secretome profiling of cytokines and growth factors in HBPCs

**Fig. 5:**
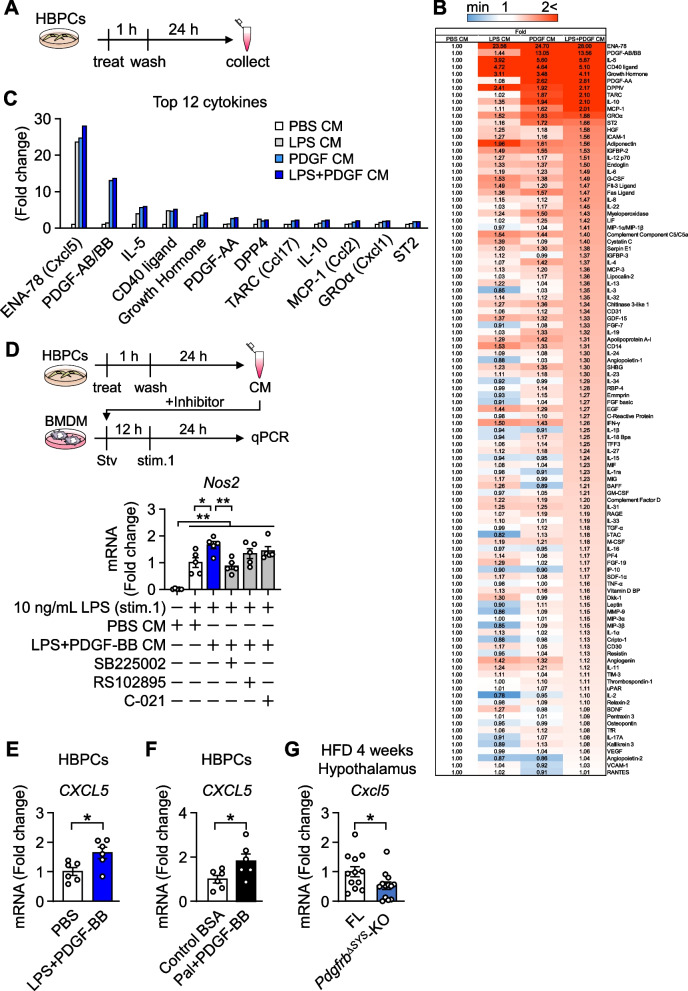
Secretome profile of HBPCs. **A** Timeline of CM collection. HBPCs were treated with LPS and/or PDGF-BB for 1 h, washed, cultured for 24 h, and CMs were collected. **B** Heat map of different protein levels in HBPC CM. The secreted levels of each factor are expressed as fold changes from the amount secreted in PBS CM. Colors from blue to red indicate a low to high protein level. **C** The top 12 proteins secreted in response to the stimulation are summarized in the graph. **D** Effects of inhibitors on the inflammatory priming of BMDMs by the pericyte secretome. BMDMs were pre-incubated with 1 μM SB225002 (CXCR1/2 inhibitor), 5 μM RS102895 (CCR2 inhibitor), or 100 nM C021 (CCR4 inhibitor) in serum-free RPMI 1640 media for 1 h, followed by a treatment with PBS CM or LPS + PDGF-BB CM for 12 h. Cells were then stimulated with 10 ng/mL LPS for 24 h, and *Nos2* expression was measured as an index of inflammatory priming (n = 5). **E** mRNA level of *CXCL5* in HBPCs treated with 100 ng/mL LPS and 100 ng/mL recombinant human PDGF-BB for 3 h (n = 6). **F** mRNA level of *CXCL5* in HBPCs treated with 100 μM Palmitate (Pal) and 100 ng/mL recombinant human PDGF-BB for 6 h (n = 6). **G** *Cxcl5* mRNA expression in the hypothalamus of FL and *Pdgfrb*^∆SYS^-KO fed HFD for 4 weeks (n = 12–13). Data are presented as the mean ± SEM. *p < 0.05 and **p < 0.01, significantly different between each group

We examined pericyte CM utilizing the cytokine array kit to investigate the pericyte-derived secretory factors that drive activation of BMDMs following stimulations with LPS and PDGF-BB. The overall secretome profiles in each CM and the top 12 factors that increased in response to the LPS and/or PDGF-BB stimulation are shown in Fig. 5A–C. Pericytes secreted various chemokines, cytokines, and growth factors in response to LPS and/or PDGF-BB. We screened the relevant factors in LPS + PDGF-BB CM that enhanced inflammatory priming in BMDMs among the top 12 factors. Epithelial-derived neutrophil-activating peptide 78 (ENA-78; C-X-C motif chemokine ligand (CXCL)5), Growth-regulated oncogene α (GROα; CXCL1), Thymus and activation-regulated chemokine (TARC; C–C motif chemokine ligand (CCL)17), and Monocyte chemoattractant protein 1 (MCP-1; CCL2) as their corresponding receptors CXCR1, CXCR2, CCR4 and CCR2, respectively, are associated with chronic inflammation and metabolic disorders (Chavey et al. 2009; Boro and Balaji 2017; Fülle et al. 2018; He et al. 2016). Therefore, we investigated the effects of inhibitors of these receptors on the LPS + PDGF-BB CM-mediated potentiation of LPS-induced inflammatory priming in BMDMs by measuring *Nos2* expression as an index (Fig. 5D). LPS-induced *Nos2* expression was significantly higher in BMDMs cultured with LPS + PDGF-BB CM than with PBS CM (lane 2 vs 3). Importantly, the expression was suppressed by SB225002, a CXCR inhibitor (lane 3 vs 4), but not by inhibitors for CCR2 and CCR4. Consistent to the pericyte secretome profile, co-stimulation with LPS and PDGF-BB augmented mRNA expression of *CXCL5* in HBPCs (Fig. 5E). The increase was also observed when HBPCs were co-stimulated with palmitate and PDGF-BB (Fig. 5F). The expression was consistently lower in the hypothalamus of *Pdgfrb*^∆SYS^-KO mice than in control FL mice following 4 weeks of HFD feeding (Fig. 5G). These results suggest that pericyte-derived CXCLs, especially CXCL5, are the most promising factors enhancing the inflammatory priming of macrophages via CXCRs.

## Discussion

**Fig. 6:**
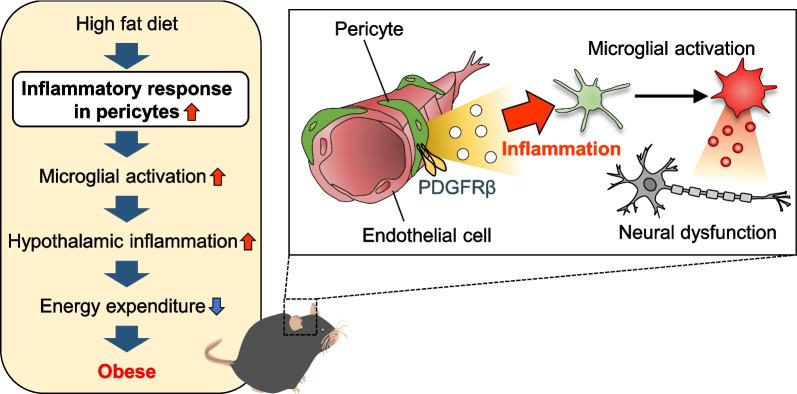
Overview of the mechanism underlying chronic hypothalamic inflammation by pericyte-microglial interactions in early obesity. A presumed mechanism of obesity based on the present study. Upon HFD, pericytes receive inflammatory inputs in addition to PDGF signaling, which changes their secretome to an inflammatory phenotype. Secreted factors mobilize microglial polarization and enhance chronic inflammation in the hypothalamus. Inflammation modulates neuronal activity and decreases energy expenditure to further promote obesity

Pericytes plays key roles in maintaining vascular homeostasis and regulating inflammatory responses in the CNS (Rustenhoven et al. 2017). Although their importance and impact on metabolic disturbance in obesity remain unknown, recent evidence suggests proinflammatory aspects of pericytes in the BBB under several disease conditions (Gaceb et al. 2018; Smyth et al. 2018). The present study revealed a critical role for PDGF-BB/PDGFRβ signaling in pericytes in the development of chronic inflammation in the hypothalamus during the early stage of obesity. PDGF signaling in pericytes shifted cellular metabolism towards the glycolytic pathway, enhanced LPS-stimulated inflammatory responses, and changed their secretome to the inflammatory phenotype. Secreted factors induced microglial priming and promoted chronic inflammation in the hypothalamus, which disrupted energy homeostasis possibly by attenuating POMC neural activity in the early stage of obesity development (Fig. 6).

The pericyte secretome induced polarization of BMDMs in vitro (Fig. 3). Adipose tissue pericytes were previously shown to secrete higher levels of pro-inflammatory factors in obese subjects than in lean subjects (Pellegrinelli et al. 2014). Similarly, PDGFRβ-positive fibro-inflammatory progenitor cells induced the inflammatory polarization of adipose tissue macrophages in obese mice (Shan et al. 2020). Pericytes have also been shown to convey early inflammatory signals to various immune cells in acute inflammation (Liu et al. 2016). In the present study, PDGF signaling did not directly affect inflammatory cytokine expression in BMDMs (Fig. 3). Moreover, proinflammatory gene expression in eWAT was indistinguishable between FL and *Pdgfrb*^∆SYS^-KO after 4 weeks of HFD feeding (Additional file 1: Fig. S1), suggesting that enhanced energy expenditure in *Pdgfrb*^∆SYS^-KO on HFD is independent of obesity-associated inflammation in adipose tissues. We concluded that this effect in *Pdgfrb*^∆SYS^-KO on HFD was due to the inhibition of pericyte-induced microglial activation and chronic inflammation in the hypothalamus (Figs. 1, 2). Microglial priming by brain pericytes appears to be a novel pathological mechanism in the early stage of obesity.

Intracellular metabolism for energy production is affected by the inflammatory activity of immune cells (Onogi et al 2020; Chou et al. 2022; Meng et al. 2021). We herein demonstrated that the PDGF-BB stimulation promoted glycolytic metabolism, which was coupled with the activation of MAPK and mTOR signals in HBPCs (Fig. 4). Therefore, similar to immune cells, PDGF-BB-induced glycolytic metabolism in pericytes appeared to be closely associated with their inflammatory secretome.

The LPS-induced inflammatory response in pericytes was enhanced by PDGF-BB and accompanied by the secretion of various cytokines and chemokines (Figs. 4, 5). The importance of the CXCR signaling was suggested by experiments with an inhibitor of the receptor against factors reported to activate microglia among the top 12 factors of the pericyte secretome. CXCL5 secreted from pericytes has been shown to promote neutrophil migration (Liu et al. 2016), and stimulated microglial proliferation during the restoration process from optic nerve inflammation (Liu et al. 2021). In addition, CXCL1 and CXCR1/2 were also found to mediate macrophage infiltration (Wang et al. 2022) and activate the NLR family pyrin domain-containing 3 inflammasome in macrophages (Boro and Balaji 2017). Therefore, it is possible that not only CXCL5 but also other CXCLs secreted from pericytes cooperate to promote hypothalamic inflammation.

Chronic inflammation precedes in the hypothalamus than in peripheral tissues during the development of obesity (Thaler et al 2012). So far, both LPS and long-chain fatty acids, which increase with obesity, have been thought to activate TLR4 expressed on microglia in the induction of hypothalamic chronic inflammation (Sheikh et al 2020; Mendes et al. 2018; Le Thuc et al. 2017) as the underlying mechanism. In the current study, we proposed an alternative mechanism that drives microglial polarization via pericytes. It has been reported that 4 weeks of HFD feeding increases serum concentration of LPS 2 to threefold, and that mice continuously administered LPS exhibit weight gain and glucose intolerance due to chronic inflammation in peripheral tissues (Cani et al. 2007). In addition, an association between high-fat diet intake and circulating LPS has also been reported in human (Amar et al. 2008). In the present study, we observed that the expression of *CXCL5*, the most secreted factor of pericytes by co-stimulation with PDGF-BB and LPS, was also increased by co-stimulation with PDGF-BB and palmitic acid (Fig. 5). Since microglial activation as well as *Cxcl5* mRNA level was consistently lower in the hypothalamus of *Pdgfrb*^∆SYS^-KO than in FL, free fatty acids may also contribute to pericyte-mediated hypothalamic inflammation in obesity. Precise biological responses between free fatty acids and PDGF-BB in pericytes need to be further explored in the future study.

BMDMs have been widely used to analyze microglial phenotypes in vitro, as their phenotype is essentially similar to that of primary microglia, especially in the inflammatory response (Jang et al 2016; Kim et al. 2018). However, microglia are cells resident in the central nervous system and are not exactly the same as BMDMs. Therefore, we have at least confirmed that pericyte CM induces morphological activation in rat primary microglia (Additional file 1: Fig. S3). Clarification of the inflammatory polarization in actual microglia by pericyte secretome is needed in the future study.

Previous studies have shown the mechanism by which chronic inflammation in the hypothalamus associated with obesity further promotes the pathology. Hypothalamic neurons, such as POMC and AgRP neurons, respond to inputs from peripheral tissues such as insulin, leptin, incretin, as well as nutrients including glucose and lipids, and contribute to systemic homeostasis by regulating feeding and energy metabolism (Tran et al. 2022; Biglari et al 2021). Chronic inflammation triggered by iNOS expression in microglia disrupts the sensing of metabolic feedback from the periphery in the POMC neuron of the hypothalamus (Lee et al. 2018; Valdearcos et al. 2017; Jais and Brüning 2017). In addition, attenuation of hypothalamic output signals that induce lipolysis in WAT and UCP1-mediated thermogenesis in BAT promotes obesity (Tran et al. 2022; Manceau et al. 2020). Since microglial activation associated with HFD loading was lower in *Pdgfrb*^∆SYS^-KO than in FL mice, energy metabolism appeared to be maintained by preserved POMC neuronal activity in the ARC and UCP1 expression in BAT (Figs. 1, 2).

PDGFRβ is expressed exclusively in pericytes/VSMCs and in some neurons (Sil et al. 2018; Crouch et al. 2023). *Pdgfrb*^∆SYS^-KO on HFD exhibited increased POMC neural activity in the ARC and UCP1 protein levels in BAT (Fig. 2). In contrast, *Pdgfrb*^∆CaMKII^-KO on HFD showed no changes in body weight or energy expenditure (Fig. 1). Since CaMKIIa expression is predominantly localized to excitatory neurons, PDGFRβ signaling in these neurons is not involved in their regulation. However, as a limitation of the current study, we did investigate the contribution of PDGFRβ in CaMKIIa-negative neurons.

Impairments in pericyte vascular coverage in the brain causes BBB dysfunctions, extravasation, and enhanced inflammation, which have been suggested as the mechanisms responsible for disease progression in Alzheimer’s disease, motor neuron diseases, and stroke (Sweeney et al. 2018; Kaushik et al. 2021; Shen et al. 2019). Pericyte dysfunction was also found to induce inflammation and pathological changes in a diabetic retinopathy model (Coucha et al. 2019; Park et al. 2017). In previous studies, congenital PDGFRβ mutant mice exhibited BBB disruption (Crouch et al. 2023). In contrast, *Pdgfrb*^∆SYS^-KO in the present study are tamoxifen-inducible gene-deficient mice and did not exhibit hyperpermeability in the hypothalamus (Additional file 1: Fig. S2). The discrepancy is recently reported similarly that PDGFB ablated mice in adult showed weaker vascular phenotypes than in congenital ablated mice (Vazquez-Liebanas et al. 2022). Therefore, although PDGF signaling is important for the physiological functions of pericytes, vascular permeability in the brain does not account for the metabolic phenotypes in *Pdgfrb*^∆SYS^-KO.

In conclusion, PDGFRβ signaling in hypothalamic pericytes promoted microglial polarization and contributed to the progression of the obesity pathology. The pericyte secretome, including CXCL5, may be a key mediator of microglial activation, which is closely associated with the suppression of energy expenditure.

The present results provide mechanistic insights into the immune responses of pericytes, and are expected to contribute to the development of novel interventional strategies for metabolic disorders and their complications.

### Supplementary Information

**Additional file 1.** Supplementary Figures and Tables.

## Data Availability

The datasets used and/or analyzed during the current study are available from the corresponding author on reasonable request.

## Abbreviations

ARC: Arcuate nucleus
AgRP: Agouti-related protein
BAT: Brown adipose tissue
BBB: Blood–brain barrier
BMDMs: Bone marrow-derived macrophages
BSA: Bovine serum albumin
CaMKII: Calcium/calmodulin-dependent protein kinase type II
CCL: C–C motif chemokine ligand
CCR: C–C motif chemokine receptor
cFOS: Cellular oncogene fos
CM: Conditioned medium
CNS: Central nervous system
CXCL: C-X-C motif chemokine ligand
CXCR: C-X-C motif chemokine receptor
DMEM/F-12: Dulbecco's Modified Eagle Medium/Ham's F-12
ECAR: Extracellular acidification rate
ENA78: Epithelial-derived neutrophil-activating peptide 78
eWAT: Epididymal white adipose tissue
FBS: Fetal bovine serum
FCCP: Carbonyl cyanide-4 (trifluoromethoxy) phenylhydrazone
FL: *Pdgfrb*^Flox^^/^^flox^ mice
GROα: Growth-regulated oncogene α
HBPCs: Human brain pericytes
HFD: High-fat diet
iWAT: Inguinal white adipose tissue
LPS: Lipopolysaccharide
MAPK: Mitogen-activated protein kinase
MCP1: Monocyte chemoattractant protein 1
mTOR: Mammalian target of rapamycin
NF-κB: Nuclear factor-κB
OCR: Oxygen consumption rate
Oligo: Oligomycin
PBS: Phosphate-buffered saline
PDGF: Platelet-derived growth factor
PDGFRβ: Platelet-derived growth factor receptor β
*Pdgfrb*^∆SYS^-KO: Conditional systemic *Pdgfrb* knockout mice
*Pdgfrb*^∆CaMK^^II^-KO: CaMKIIa-positive neuron-specific *Pdgfrb* knockout mice
PFA: Paraformaldehyde
POMC: Proopiomelanocortin
Rot/AA: A mix of rotenone/antimycin A.
SEM: Standard error of the mean
TARC: Thymus and activation-regulated chemokine
TLR4: Toll-like receptor 4
UCP1: Uncoupling protein 1
VMH: Ventromedial hypothalamic nucleus
VSMCs: Vascular smooth muscle cells
