# Supplementary material for: Platelet-derived growth factor signaling in pericytes promotes hypothalamic inflammation and obesity
Source: Mol Med. 2024 Feb 5;30:21. doi: 10.1186/s10020-024-00793-z (PMC10845801; doi:10.1186/s10020-024-00793-z)
Supplement: Supplementary file 1 — Additional file 1. Supplementary Figures and Tables. [file 10020_2024_793_MOESM1_ESM.pptx]

## Slide 1
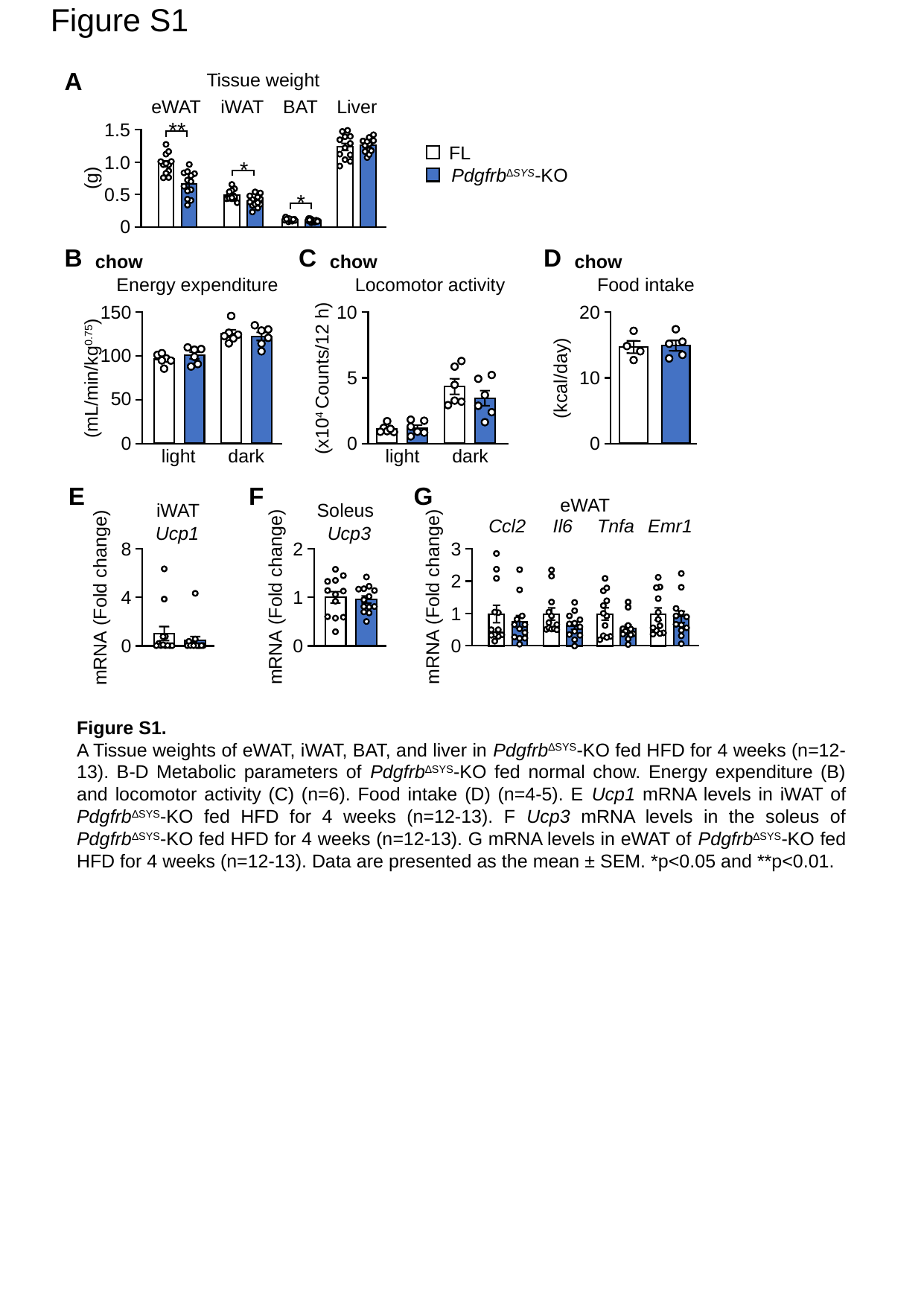

Figure S1
Tissue weight
A
eWAT
iWAT
BAT
Liver
**
1.5
FL
1.0
*
Pdgfrb∆SYS-KO
(g)
0.5
*
0
B
C
D
chow
chow
chow
Energy expenditure
Locomotor activity
Food intake
150
10
20
100
5
10
(mL/min/kg0.75)
(x104 Counts/12 h)
(kcal/day)
50
0
0
0
light
dark
light
dark
E
F
G
eWAT
iWAT
Soleus
Ccl2
Il6
Tnfa
Emr1
Ucp1
Ucp3
3
8
2
2
mRNA (Fold change)
mRNA (Fold change)
4
1
mRNA (Fold change)
1
0
0
0
Figure S1.
A Tissue weights of eWAT, iWAT, BAT, and liver in Pdgfrb∆SYS-KO fed HFD for 4 weeks (n=12-13). B-D Metabolic parameters of Pdgfrb∆SYS-KO fed normal chow. Energy expenditure (B) and locomotor activity (C) (n=6). Food intake (D) (n=4-5). E Ucp1 mRNA levels in iWAT of Pdgfrb∆SYS-KO fed HFD for 4 weeks (n=12-13). F Ucp3 mRNA levels in the soleus of Pdgfrb∆SYS-KO fed HFD for 4 weeks (n=12-13). G mRNA levels in eWAT of Pdgfrb∆SYS-KO fed HFD for 4 weeks (n=12-13). Data are presented as the mean ± SEM. *p<0.05 and **p<0.01.

## Slide 2
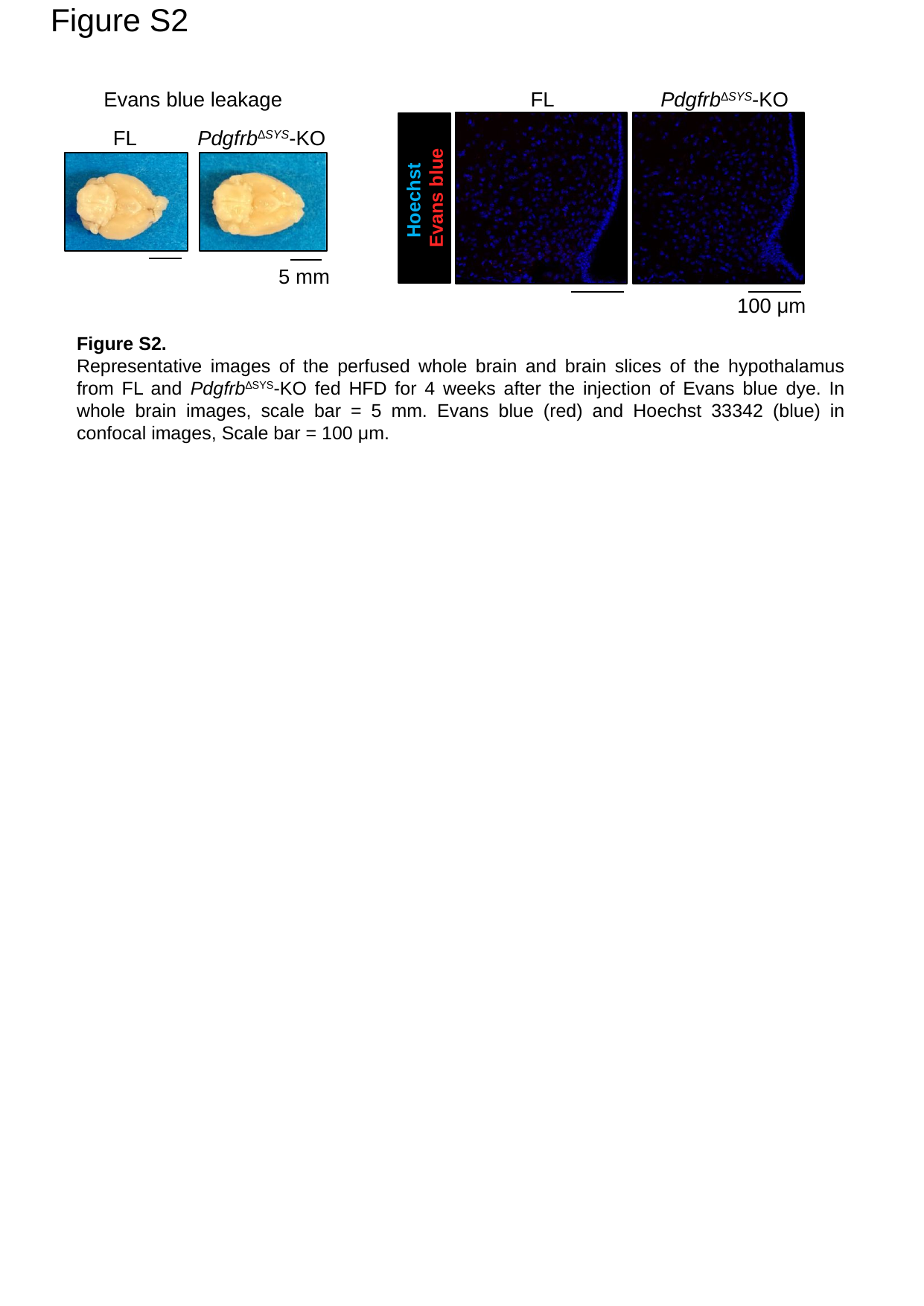

Figure S2
Evans blue leakage
FL
Pdgfrb∆SYS-KO
Hoechst
Evans blue
FL
Pdgfrb∆SYS-KO
5 mm
100 μm
Figure S2.
Representative images of the perfused whole brain and brain slices of the hypothalamus from FL and Pdgfrb∆SYS-KO fed HFD for 4 weeks after the injection of Evans blue dye. In whole brain images, scale bar = 5 mm. Evans blue (red) and Hoechst 33342 (blue) in confocal images, Scale bar = 100 μm.

## Slide 3
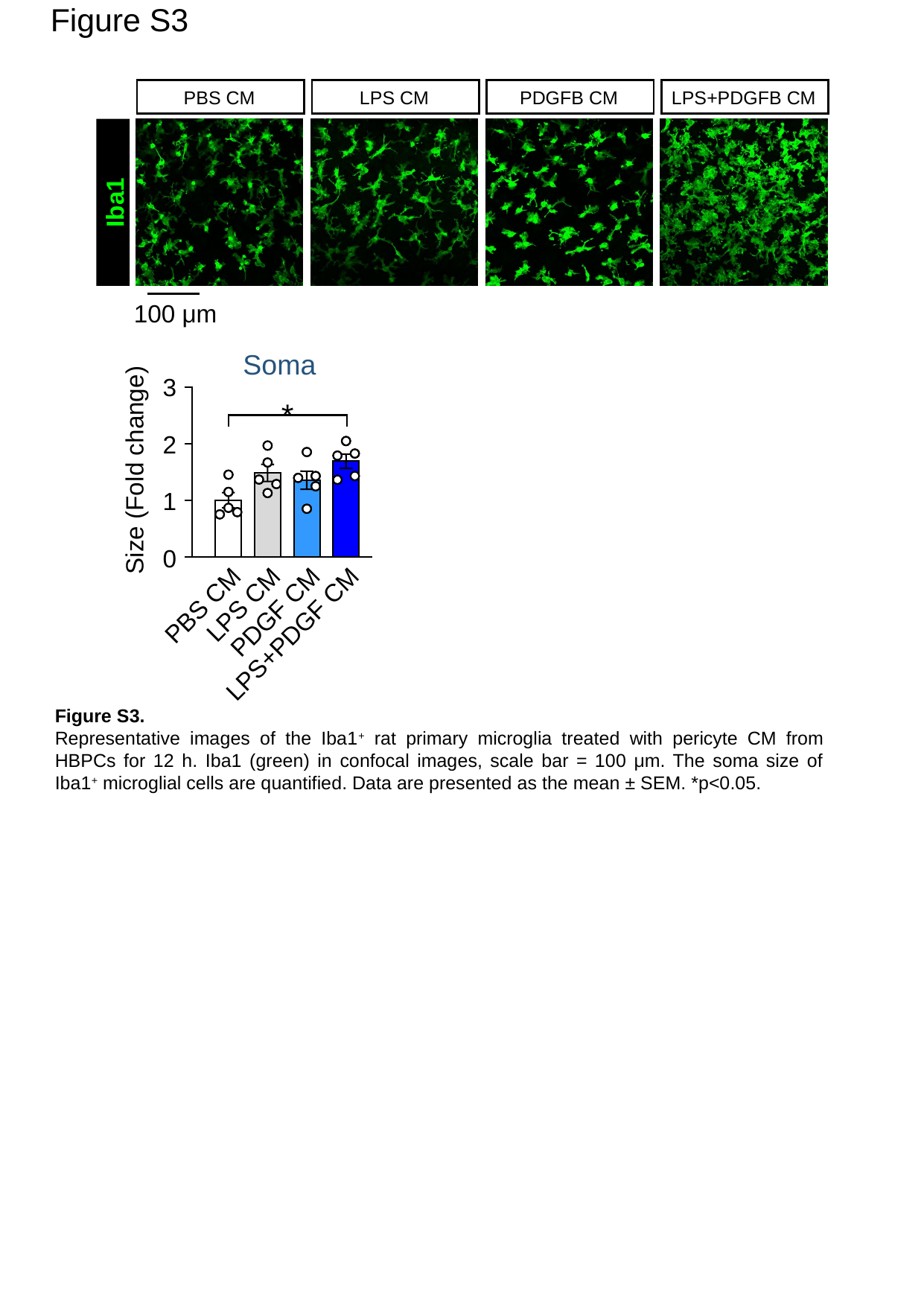

Figure S3
PBS CM
LPS CM
PDGFB CM
LPS+PDGFB CM
Iba1
100 μm
Soma
3
*
2
Size (Fold change)
1
0
PBS CM
PDGF CM
LPS CM
LPS+PDGF CM
Figure S3.
Representative images of the Iba1+ rat primary microglia treated with pericyte CM from HBPCs for 12 h. Iba1 (green) in confocal images, scale bar = 100 μm. The soma size of Iba1+ microglial cells are quantified. Data are presented as the mean ± SEM. *p<0.05.

## Slide 4
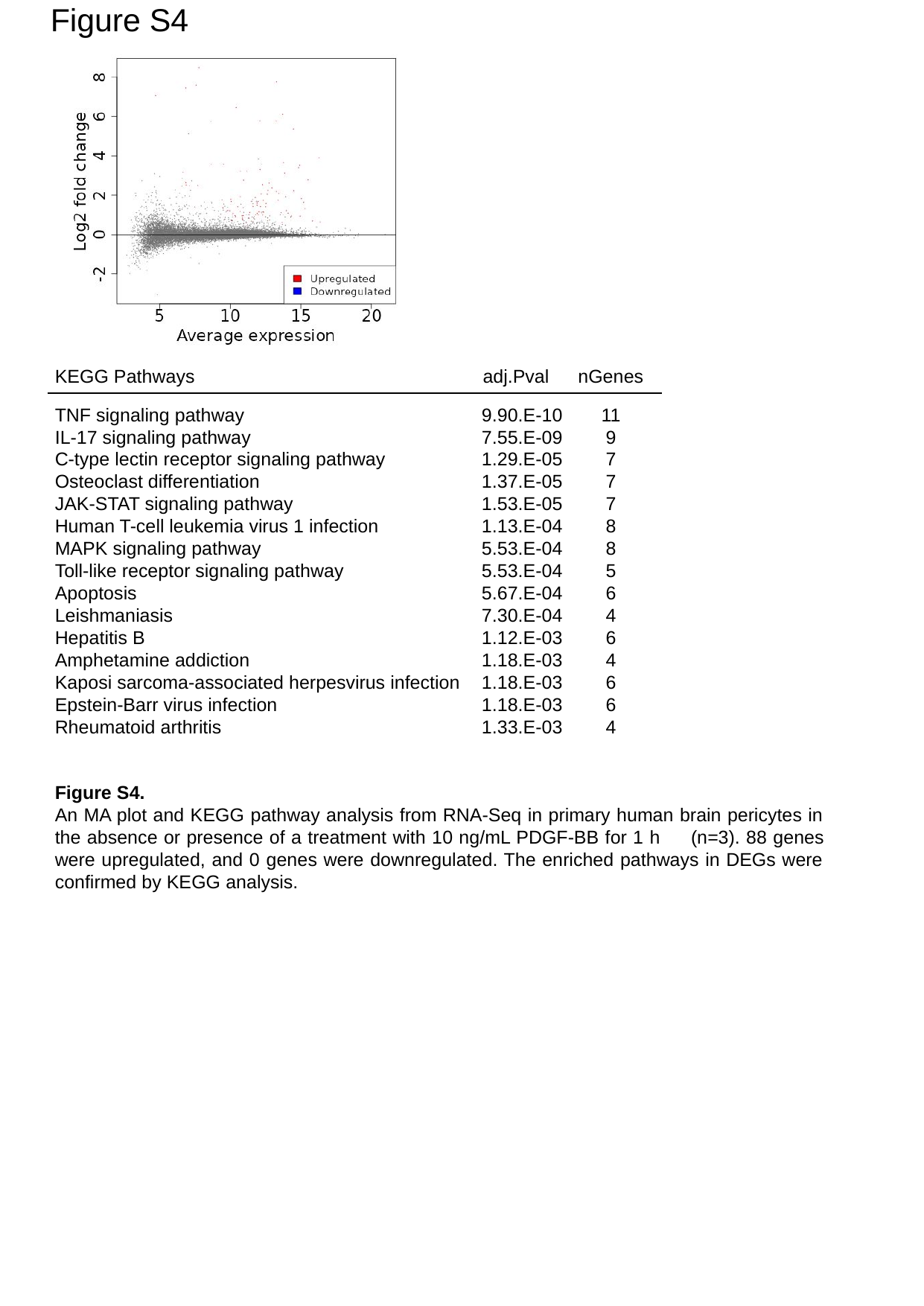

Figure S4
KEGG Pathways
adj.Pval
nGenes
TNF signaling pathway
IL-17 signaling pathway
C-type lectin receptor signaling pathway
Osteoclast differentiation
JAK-STAT signaling pathway
Human T-cell leukemia virus 1 infection
MAPK signaling pathway
Toll-like receptor signaling pathway
Apoptosis
Leishmaniasis
Hepatitis B
Amphetamine addiction
Kaposi sarcoma-associated herpesvirus infection
Epstein-Barr virus infection
Rheumatoid arthritis
9.90.E-10
7.55.E-09
1.29.E-05
1.37.E-05
1.53.E-05
1.13.E-04
5.53.E-04
5.53.E-04
5.67.E-04
7.30.E-04
1.12.E-03
1.18.E-03
1.18.E-03
1.18.E-03
1.33.E-03
11
9
7
7
7
8
8
5
6
4
6
4
6
6
4
Figure S4.
An MA plot and KEGG pathway analysis from RNA-Seq in primary human brain pericytes in the absence or presence of a treatment with 10 ng/mL PDGF-BB for 1 h　(n=3). 88 genes were upregulated, and 0 genes were downregulated. The enriched pathways in DEGs were confirmed by KEGG analysis.

## Slide 5
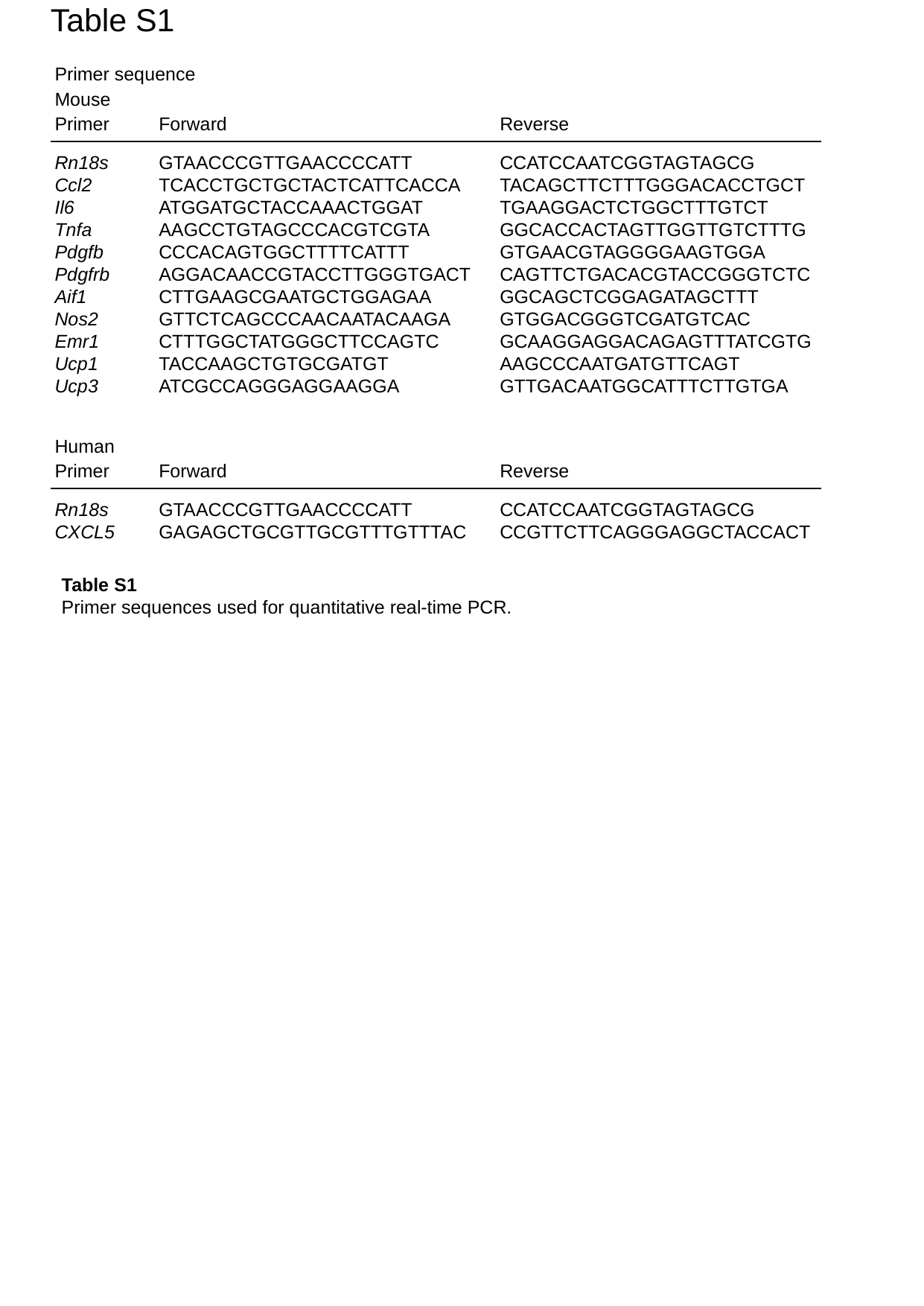

Table S1
Primer sequence
Mouse
Primer
Forward
Reverse
Rn18s
Ccl2
Il6
Tnfa
Pdgfb
Pdgfrb
Aif1
Nos2
Emr1
Ucp1
Ucp3
GTAACCCGTTGAACCCCATT TCACCTGCTGCTACTCATTCACCA
ATGGATGCTACCAAACTGGAT
AAGCCTGTAGCCCACGTCGTA
CCCACAGTGGCTTTTCATTT
AGGACAACCGTACCTTGGGTGACT
CTTGAAGCGAATGCTGGAGAA
GTTCTCAGCCCAACAATACAAGA
CTTTGGCTATGGGCTTCCAGTC
TACCAAGCTGTGCGATGT
ATCGCCAGGGAGGAAGGA
CCATCCAATCGGTAGTAGCG TACAGCTTCTTTGGGACACCTGCT
TGAAGGACTCTGGCTTTGTCT
GGCACCACTAGTTGGTTGTCTTTG
GTGAACGTAGGGGAAGTGGA
CAGTTCTGACACGTACCGGGTCTC
GGCAGCTCGGAGATAGCTTT
GTGGACGGGTCGATGTCAC
GCAAGGAGGACAGAGTTTATCGTG
AAGCCCAATGATGTTCAGT
GTTGACAATGGCATTTCTTGTGA
Human
Primer
Forward
Reverse
Rn18s
CXCL5
GTAACCCGTTGAACCCCATT
GAGAGCTGCGTTGCGTTTGTTTAC
CCATCCAATCGGTAGTAGCG
CCGTTCTTCAGGGAGGCTACCACT
Table S1
Primer sequences used for quantitative real-time PCR.

## Slide 6
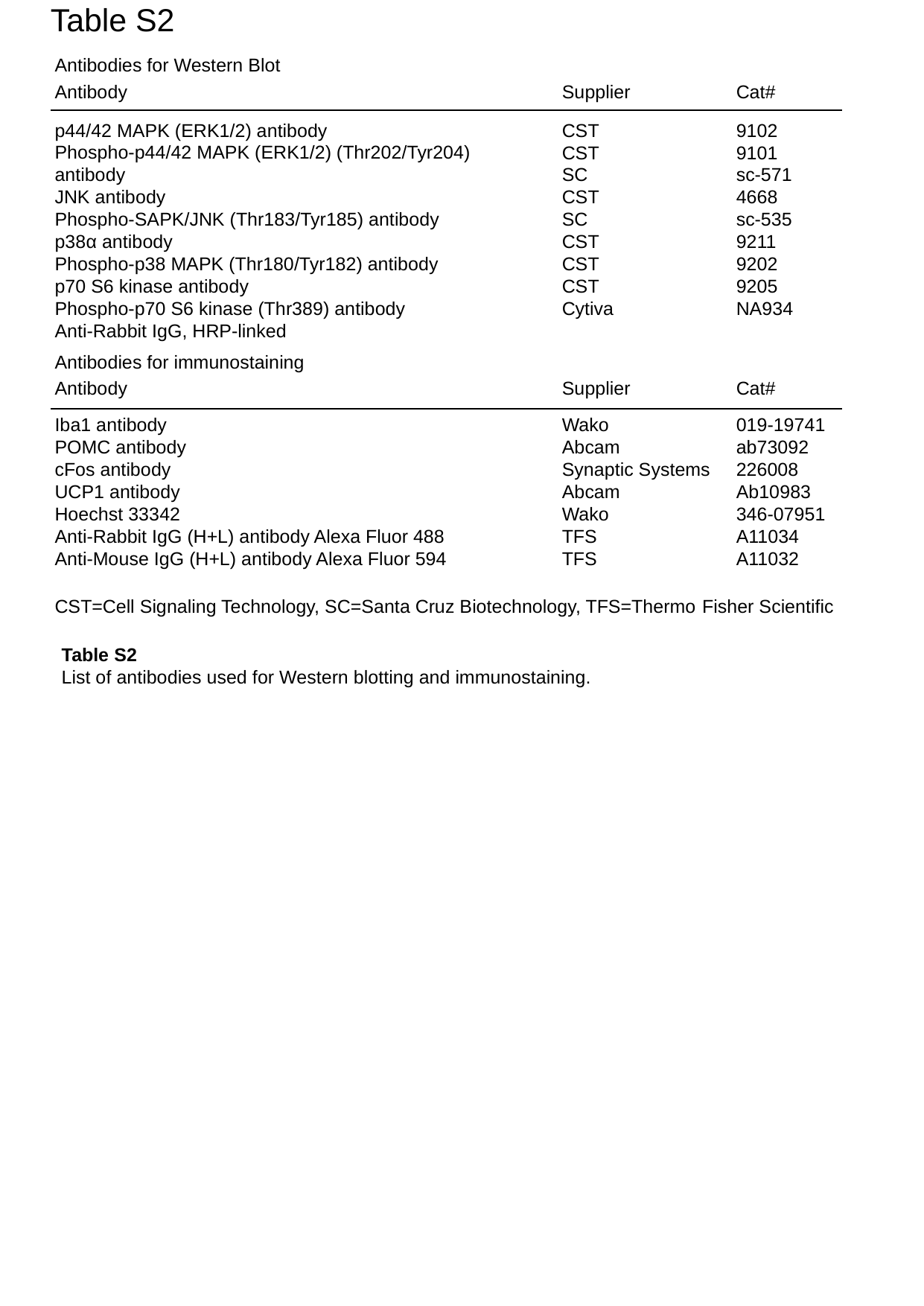

Table S2
Antibodies for Western Blot
Antibody
Supplier
Cat#
p44/42 MAPK (ERK1/2) antibody
Phospho-p44/42 MAPK (ERK1/2) (Thr202/Tyr204) antibody
JNK antibody
Phospho-SAPK/JNK (Thr183/Tyr185) antibody
p38α antibody
Phospho-p38 MAPK (Thr180/Tyr182) antibody
p70 S6 kinase antibody
Phospho-p70 S6 kinase (Thr389) antibody
Anti-Rabbit IgG, HRP-linked
CST
CST
SC
CST
SC
CST
CST
CST
Cytiva
9102
9101
sc-571
4668
sc-535
9211
9202
9205
NA934
Antibodies for immunostaining
Antibody
Supplier
Cat#
Iba1 antibody
POMC antibody
cFos antibody
UCP1 antibody
Hoechst 33342
Anti-Rabbit IgG (H+L) antibody Alexa Fluor 488
Anti-Mouse IgG (H+L) antibody Alexa Fluor 594
Wako
Abcam
Synaptic Systems
Abcam
Wako
TFS
TFS
019-19741
ab73092
226008
Ab10983
346-07951
A11034
A11032
CST=Cell Signaling Technology, SC=Santa Cruz Biotechnology, TFS=Thermo Fisher Scientific
Table S2
List of antibodies used for Western blotting and immunostaining.
